# Urinary 1-hydroxypyrene and Skin Contamination in Firefighters Deployed to the Fort McMurray Fire

**DOI:** 10.1093/annweh/wxz006

**Published:** 2019-02-08

**Authors:** Nicola Cherry, Yayne-abeba Aklilu, Jeremy Beach, Philip Britz-McKibbin, Rebecca Elbourne, Jean-Michel Galarneau, Biban Gill, David Kinniburgh, Xu Zhang

**Affiliations:** 1 Division of Preventive Medicine, Department of Medicine, University of Alberta, 5–22 University Terrace, Edmonton, Alberta, Canada; 2 Alberta Environment and Parks, Edmonton, Alberta, Canada; 3 Department of Chemistry and Chemical Biology, McMaster University, Hamilton, Ontario, Canada; 4 Alberta Centre for Toxicology, Calgary, Alberta, Canada

**Keywords:** firefighter, 1-hydroxypyrene, polycyclic aromatic hydrocarbons, skin absorption

## Abstract

**Background:**

In May 2016, firefighters from the province of Alberta, Canada deployed to a fire that engulfed the urban area of Fort McMurray. During the first days of the fire, firefighters experienced heavy smoke exposures during greatly extended work shifts. Urinary samples were collected post-deployment from three fire services for estimation of 1-hydroxypyrene (1-HP) concentration, reflecting exposure to polycyclic aromatic hydrocarbons (PAHs), to determine the effects of respiratory protective equipment (RPE) and skin hygiene in reducing internal dose

**Methods:**

Urine samples from one fire service (*n* = 62) were analyzed for 1-HP by two laboratories, using different assays (LC-MS/MS: GC-MS): remaining samples were analyzed just by LC-MS/MS. A Skin Exposure Mitigation Index (SEMI) was computed from questions on opportunities for changing clothing, showering, and washing during breaks. Regression analyses, using 1-HP ng/g creatinine as the dependent variable, assessed the effect of RPE and skin factors on PAH absorption, allowing for environmental exposure and potential confounders. Stratification identified key groups with equal delay in sample collection.

**Results:**

1-HP was detected in 71.0% of 62 samples by LC-MS/MS and 98.4% by GC-MS, with good mutual agreement between the methods. In 171 post-fire samples, 1-HP corrected for creatinine was related to current cigarette smoking and recent barbeque. Among those with samples collected within 48 h, urinary 1-HP was correlated with estimated exposure(*r* = 0.53, *P* < 0.001). In those with only one rotation before urine sample collection, no effect was seen of RPE use but I-HP was significantly lower (*P* = 0.003) in those with those with a high score on the SEMI scale, indicating better access to factors mitigating skin absorption.

**Conclusion:**

Skin exposure to PAHs is an important route of absorption in firefighters, which can be mitigated by good skin hygiene.

## Introduction

On 2 May 2016, a wildfire threatened to engulf the urban area of Fort McMurray in the north of the province of Alberta, Canada, resulting in a near-total evacuation. Firefighters were deployed from across the province and beyond to help control the fire during May and June 2016. It was soon recognized that the extreme conditions early in the fire could affect the health of first responders. To investigate this, indices of exposure and effect would ideally have been collected immediately post-deployment but no research access to the area by road was possible during the early weeks of the fire. Recently exposed firefighters were recruited from two sources. The first was a large fire service located close to Edmonton, where the Fire Chief agreed that all firefighters who had been deployed (and returned) by mid-May could be offered the chance to participate in the study. The second was a group of industrial firefighters located north of Fort McMurray and who were reached by a company plane in early June. It was important to include also firefighters employed by the Fort McMurray fire service, but it did not prove possible to recruit them until some 4 months after the start of the fire. Firefighters from these three services completed questionnaires about their exposures, ability to keep skin clean and use of respiratory protective equipment (RPE) during their first deployment and about their health immediately before and after the fire. They also gave a spot urine sample. Ten other fire services (for a total of 355 firefighters) also took part in this phase of the study but were not asked for biological samples.

During the first few days of the fire, conditions were very severe, with many firefighters working for 24 h or more, catching sleep where they could in their vehicle and with food limited to emergency rations eaten in the field during minimal breaks. The supply and use of RPE was difficult. Many of the firefighters had no access to clean clothes or to showers and had no facilities to wash during food breaks. All of these conditions improved during the first week, with greater availability of RPE and arrangements for cots, showers, and hot meals.

A number of recent studies of firefighters exposed during prescribed burns (controlled fires used in training or forest management) have examined urinary metabolites, collected immediately post-exposure, as a biomarker for polycyclic aromatic hydrocarbons (PAHs) exposure (Fent *et al*., 2014; Fernando *et al*., 2016; Adetona *et al*., 2017; Keir *et al*., 2017; Oliveira *et al*., 2017; Stec *et al*., 2018; Wingfors *et al*., 2018). We are not aware of studies examining PAH metabolites in firefighters engaged in an extensive and prolonged uncontrolled structural fire such as in Fort McMurray or where urinary samples have been collected after days or weeks after exposure. The American Conference of Governmental Hygienists (ACGIH) recommends 1-hydroxypyrene (1-HP) as the biological exposure index for PAH exposure (ACGIH, 2017), with samples collected at the end of shift, at the end of the work week. 1-HP urinary concentration reflects exposure to pyrenes (not themselves carcinogenic) together with more hazardous PAHs including benzo(a) pyrene, classified as a human carcinogen by the International Agency for Research on Cancer (IARC, 2012).

There is some uncertainty about the half-life of 1-HP. Jongeneelen *et al*. (1988) observed (in one worker) a fast-excreting component with a half-life of 1–2 days and a slow excreting component with a half-life of 16 days. There do not appear to be any attempts to replicate this observation of a multiphasic pattern although Bouchard *et al*. (2002) reported a bi-phasic pattern in rats, with 1-HP taking 9 days to return to pre-dosing levels. Viau *et al*. (1995) suggested that a two-compartment model was needed to explain excretion patterns in exposed volunteers. The ACGIH (2017) BEI documentation for PAHs tabulates half-lives of urinary 1-HP from the literature, with the shortest <4 h and the longest (other than Jongeneelen *et al*.) 108 h. Skin absorption appears to result in a later peak of urinary excretion than pulmonary absorption (Lafontaine *et al*., 2000) with continued absorption through contaminated clothing (Lafontaine, 2002).

In the present study, use of a biomarker such as 1-HP was particularly appropriate as it integrates exposures by inhalation, dermal exposure and ingestion, all likely exposure routes during the early days of the fire. The analysis reported here attempted to use urinary 1-HP, collected at different intervals post-exposure, to examine the relation between estimated environmental exposures, use of RPE and factors mitigating the potential for exposure through skin or oral ingestion. The aim was to identify ways in which the internal dose of PAHs could be reduced in future engagements.

## Methods

### Three fire services were included as the study population

Fire service A was a large fire service close to Edmonton that deployed many firefighters to the Fort McMurray fire, using rapidly rotating shifts to limit the exposure of individual firefighters. They were among the best equipped and organized of the crews that attended. Some, but not all, of those deployed were exposed under the more chaotic conditions early in the fire. Most had been deployed only once at the first visit of the research team (May 16–20th).

Fire service B was an industrial fire service maintained by a major company in the oil and gas industry that is the mainstay of Fort McMurray’s development. These firefighters were all engaged from the very beginning, first during efforts to contain the wildfire, but then also within the burning city. Most had been deployed for several rotations by the time of study recruitment on June 6th.

Fire service C was the permanent Fort McMurray/Wood Buffalo fire service. All had been involved from the first day of the fire, and almost all served repeated rotations. They were probably less well equipped than Fire Service A and all had worked for very long hours under heavily exposed and demanding conditions early in the fire. By the time that they were recruited for the study (31st August to 30th September) all had completed their final rotation to this fire at least 37 days earlier.

At each fire service, only those present (and consenting) during the visit of the research team could be included in the study. At fire services A and B, some firefighters were not present either because they were actively engaged on duties at the fire or were on leave following deployment. At fire service C, it proved difficult to reach all eligible fire fighters.

Firefighters from all three services not reached during the first recruitment visits had later opportunities to join a more broad-based study of the effects of the fire, but not to give urine samples.

### Urine collection, storage, and analysis

At fire services A and C, spot mid-stream urine samples were collected into a sterile 100 ml plastic container within the fire hall and immediately delivered by the firefighter to the mobile clinical laboratory parked in an adjacent parking lot. The sample was aliquoted into 3 × 2 ml tubes and the residue discarded. The aliquots were stored at −80°C until dispatched for analysis. At fire service B, where road access was not possible, urine samples were stored in the 100 ml collection container at −80°C on site and transported to Edmonton for storage once travel conditions became easier.

No assay for 1-HP was readily available and it was necessary to develop and validate the method before undertaking the analysis of all samples. Initially 62 samples from firefighters from fire service A were selected for analysis in duplicate, by research laboratories at the Alberta Centre for Toxicology (University of Calgary) (DK) using LC-MS/MS and at McMaster University (PB-M) by GC-MS, using the facilities of Ontario Ministry of Environment, Conservation and Parks. Only the results of the LC-MS/MS analysis are used here with the method described (Supplementary Materials 1, available at *Annals of Occupational Hygiene* online). Where concentration was below the level of detection (LoD), it was replaced by the LoD/√2, before the correction for creatinine. Samples with creatinine < 30 or >300 mg/dL were excluded from the exposure analysis (WHO, 1996).

### Questionnaire

The research team met with the firefighters when they came to one of the fire halls to hear more about the study. The firefighter, if willing, read the information sheet, signed the consent form, and completed a written questionnaire that was administered by the team, usually with a group of participants. Those who had had more than one rotation were asked to give dates of all deployments but with detailed information only for the first, including the hours actually fighting the fire in each 24 h within that deployment and the facilities during breaks (water to wash) and between shifts (showers, clean clothes). The fire service supplied rotation schedules to help in completion of dates. Visual cues (pictures of respirators) were provided to help with questions on RPE use during each task and a map of Fort McMurray for a question about where they had been deployed. The firefighter was asked the time (5% or greater) spent on each of six named tasks (actively attacking burning fires, overhauling hotspots, backburn, patrolling, protection of unburnt areas, operating equipment/driving) with space to describe up to three additional tasks. For each task, the firefighter was asked the RPE used (type, percentage of time, frequency of changing mask or filters) and to assess the typical and the worst level of smoke and dust, using pictures previously validated by Reinhardt and Ottmar (2000). The firefighter was also asked about respiratory health immediately prior to the fire, during or immediately after the first deployment and at the time of completing the questionnaire, together with any health issues they believed had been caused or made worse by their exposures during the fire. They were asked to complete a short mental health questionnaire (Bjelland *et al*., 2002) and about their history of cigarette smoking.

### Estimate of environmental exposure

**Figure 1.**
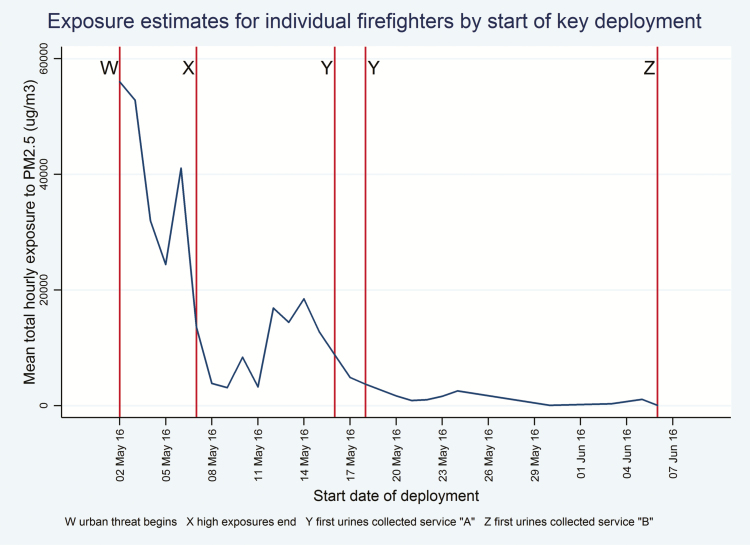
Exposure estimates for individual firefighters by start of key deployment.

Exposure to particulate matter (PM2.5) was calculated for the first deployment of each firefighter, using self-reports of dates deployed, the proportion of time spent in each area of the city and the lengths of each shift (Supplementary Materials 2, available at *Annals of Occupational Hygiene* online). Data were obtained from Alberta Environment and Parks on mean 24-h PM2.5 concentration interpolated from monitoring stations for each area of the urban site for each day May 1st to June 30th. These environmental data were used to calculate cumulative exposure across all shifts during the first deployment (Fig. 1). A factor reflecting task-specific exposures during the first deployment used the mean rating on a five-point scale from no visible smoke to very heavy smoke (Reinhardt and Ottmar, 2000) across all 355 firefighters of their typical smoke and dust levels in that task. This ranged, with weightings given by Reinhardt and Ottman, from 2.7 while patrolling to 4.1 when actively attacking the fire. The task-specific exposure factor was the time-weighted sum of such task exposures. The exposure variable used here for the first deployment is the product of these two estimates, of environmental and task-specific exposures/1000. For firefighters with more than one deployment, a total exposure summed over deployments was also calculated, based on mean 24-h PM 2.5 concentration (from Alberta Environment and Parks) for the dates of deployments but without adjustment for task-specific exposures.

### Use of respiratory protection

A use of RPE quotient was computed from self-reports of type of respiratory protective equipment used in each task, the proportion of time it was used in the task, the time spent in the task, the frequency of changing the mask, filter or cartridge, and the published protection factor (Supplementary Materials 3, available at *Annals of Occupational Hygiene* online). The quotient was summed over all tasks then adjusted for the proportion of time in each task. The resulting factor ranged from 0 to 1, with a firefighter never using respiratory protection receiving a score of zero. A firefighter who had used self-contained breathing apparatus (SCBA) correctly throughout would have received a score of 1.

### Exposure through the skin

Responses (yes/no) to three questions were used:

(i) In breaks within shift, were there facilities for you to wash?
(ii) Between shifts, were you able to shower?
(iii) Between shifts, were you able to change into clean clothes?

A ‘skin exposure mitigation index’ (SEMI) was calculated by counting the number of opportunities for better hygiene that were available. This ranged from zero (no opportunity to change clothes, shower or wash during breaks) to 3 (all possible).

### Potential confounders

Sex, age, and body mass index (BMI), calculated from measured height and weight, were considered as factors that might influence uptake, storage, metabolism or excretion of 1-HP. Current and ex-smokers were identified and the firefighter was asked if they had consumed barbequed or smoke flavored food in the 48 h before giving the sample. Firefighters were asked if they had been deployed to any fire between their last deployment to the Fort McMurray fire and the day of the urine sample collection. Fighting a different fire in the previous 3 days or in 14 days before the urine sample was considered as potential confounders.

### Statistical methods

Using 1-HPng/g creat estimated by GC-MS/MS as the dependent variable, linear regression analysis considered the relationship with potential confounders and, allowing for confounding, in a multivariable linear model, differences in 1-HP excretion among fire services. The relation of 1-HP ng/g creat to overall estimated exposure was examined by Pearson correlation for those for whom a sample had been collected within 2 days of the most recent exposure. The effects of RPE and skin hygiene measures on 1-HP ng/g creat were examined in a multivariable linear regression analysis including environmental exposure and potential confounders and in a subset of firefighters deployed during the earliest days of the fire. Mean 1-HPng/g creat and potential confounders (exposure, use of RPE, days to sample collection) were examined by skin exposure mitigation index, using analysis of variance.

### Choice of subsamples for analysis

As shown in Fig. 1, the fire first threatened the urban area of Fort McMurray on 2nd May (point W) with very high exposures to May 7th (point X). First urine samples were collected in fire service A from 16th to 18th May (point Y) and at fire service B on June 6th (point Z). Investigation of the relation between estimated exposure and 1-HPng/g creat was most readily interpretable for those for whom urine collection had been carried out within 48 h of most recent exposure (that is those exposed in 2 days before point Y or point Z). This subgroup is referred to below as Subgroup YZ. The subgroup of greatest interest, however, were those who had been deployed in the first week (in the period between point W and point X) who had the greatest exposure and it transpires (see below) the greatest opportunity for dermal exposure. This subgroup is referred to below as subgroup WX.

## Results

### Comparison of laboratory methods

The comparison of laboratory methods for the estimation of 1-HP is reported elsewhere (Gill *et al*., 2019). The GC-MS method adopted by the McMaster University group had a lower limit of detection (LoD) and somewhat higher estimates than the University of Calgary group using an LC-MS/MS method. Concentrations were above the limit of detection (LoD) for 44/62 (71.0%) by LC-MS/MS and 61/62 (98.4%) by GC-MS but there was good mutual agreement between the methods, giving credibility to the analysis reported here (Gill *et al*., 2019). Subsequent analysis of urine samples was performed by LC-MS/MS due to its faster analysis times that avoid the need for complicated sample handling.

### Analysis of samples from the three fire services

A total of 185 participants from the three services provided a urine sample with 172 of the samples having creatinine within the acceptable range (≥30 and ≤300 mg/dL). Of these 172, nine had concentrations of 1-HP below the limit of quantification and were replaced by 0.014 and 18 below the limit of detection that were replaced by 0.007.

As seen in Table 1, the great majority of the participants were male and the majority had never smoked. Those from fire service B (the industrial fire service) were less likely to be a non-smoker, were older, had a higher BMI, were more likely to have eaten barbequed food in the previous 48 h and had the highest proportion in a mainly logistic or managerial (rather than purely firefighter) role. None had been deployed at other fires but 19 of those from Strathcona and 8 from Fort McMurray had such additional exposures. Participants from the three services differed importantly on dates of first deployment to the fire. All participants from services B and C but only 44% of those from service A had been deployed during the first days of the fire, when conditions were at their worst. The large majority of those from Service A had been deployed only once to the fire at the date of sample collection but very few of those from the other services reported only a single deployment. Overall, there were only 40 samples collected within 48 h of last exposure, and none of these were from service C. The mean concentrations of urinary 1-HP for the three services are also shown in Table 1. Analysis of variance showed a significant difference in mean 1-HPng/g creat between the services (*F* = 5.5; *P* = 0.005), service B having the highest mean (99.0 ng/g creat) and service A the lowest (53.1 ng/g creat).

**Table 1.** Comparison of participants from the three fire services.

	Fire service			
	A	B	C	Overall
Male % (*N*)	97.5 (78)	94.1 (16)	90.7 (68)	94.2 (162)
Smoking				
Current smoker % (*N*)	1.3 (1)	11.8 (2)	2.7 (2)	2.9 (5)
Ex-smoker % (*N*)	11.3 (9)	52.9 (9)	17.3 (13)	18.0 (31)
Never smoked % (*N*)	87.5 (70)	35.3 (6)	80.0 (60)	79.1 (136)
Median age (range)	37.0 (22–60)	45.0 (28–54)	30.0 (21–64)	35.5 (21–64)
Median BMI (range)	28.5 (22.9–46.6)	31.2 (26.5–43.9)	28.4 (22.6–38.9)	28.7 (22.6–46.6)
Ate BBQ in last 48 h % (*N*)	32.5 (54)	70.6 (12)	36.0 (27)	37.8 (65)
Firefighter role % (*N*)	86.3 (69)	76.5 (13)	93.3 (70)	88.4 (152)
Fought a different fire in the 14 days before urine sample	23.6 (19)	0.0 (0)	10.8 (8)	15.7 (27)
Started by May 7th % (*N*)	43.8 (35)	100.0 (17)	100.0 (75)	73.8 (127)
Sample collected within 48 h of last exposure % (*N*)	38.8 (31)	52.9 (9)	0.0 (0)	23.3 (40)
Only one deployment to sample collection % (*N*)	87.5 (70)	11.8 (2)	6.7 (5)	44.8 (77)
1-HPng/g creat: mean (SD)	53.1 (55.5)	99.0 (60.0)	64.7(46.3)	62.7 (53.5)
*N*	80	17	75	172

The potential for sex, smoking, age, BMI, and eating BBQ to act as confounders was examined in a regression analysis including all participants (Table 2). With 1-HP ng/g creatinine as the dependent variable, no significant relation was seen with age, sex or BMI. Current smokers and those who had recently eaten BBQ had higher 1-HP ng/g creat, as did those who had attended another fire within 14 days of urine collection since their most recent Fort McMurray deployment. No effect of recent fires was seen when only fires within the last 3 days were included.

**Table 2.** Relation of 1-HP ng/g creat to potential confounders and fire service: multivariable analysis (*N* = 171*).

Potential confounder	Beta	SE	95% CI	*P*
Sex (male)	−24.1	15.5	−54.7 to 6.5	0.12
Age	0.1	0.4	−0.8 to 0.9	0.92
Smoking				
Current smoker	115.1	21.9	72.3 to 158.7	<0.01
Ex-smoker	18.3	10.2	−0.8 to 38.4	0.07
BMI	0.8	0.9	−1.1 to 2.6	0.41
Recent BBQ	15.2	7.7	0.04 to 30.4	0.05
Role of fire fighter	25.7	11.9	2.3 to 49.1	0.03
Fought a different fire in previous 14 days	32.6	11.4	12.0 to 53.1	<0.01
Fire Service				
B	27.0	14.2	−1.1to 66.1	0.06
C	9.3	7.9	−6.3 to 24.9	0.24

One firefighter from Fort McMurray omitted because of missing data on other fires since.

Table 3 shows the mean estimates of total particulate exposure during May and June for firefighters from each of the fire services. For first deployment only, data are shown for mean particulates, mean score on the factor reflecting smoke intensity within tasks, use of RPE and the index (SEMI) reflecting the potential for mitigating skin exposure during this first deployment. Those from Service C had the highest exposures, carried out tasks with the highest smoke density, reported the lowest mean use of RPE and the lowest potential to mitigate skin exposure. Conversely, service A had the lowest exposures, best RPE use, and highest SEMI.

**Table 3.** Mean exposures and modifying factors by fire service.

	Fire services								
	A		B		C		Overall		*P*
	Mean	SD	Mean	SD	Mean	SD	Mean	SD	
Total particulates over all deployments	13.7	14.1	56.2	23.5	77.2	17.7	45.6	34.7	<0.001
Particulates during first deployment	12.3	13.3	41.9	20.5	63.0	15.2	37.3	28.4	<0.001
Smoke intensity by tasks—first deployment	3.12	0.35	3.27	0.43	3.31	0.28	3.22	0.35	0.002
RPE use index—first deployment	0.22	0.21	0.14	0.24	0.10	0.13	0.16	0.19	<0.001
Skin index—first deployment	2.18	0.90	2.06	0.90	0.81	0.91	1.57	1.12	<0.001
*N*	80		17		75		172		

Investigating the relation between estimated exposure and 1-HP was not meaningful where the time between last exposure and sample collection was many weeks. For Fire Service C, the median time since last exposure to the fire was 71 days (and minimum 37 days) and no relation was seen between 1-HPng/g creat and either estimated total exposure over all deployments (*r* = −0.14; *P* = 0.23) or days since last exposure (*r* = 0.17; *P* = 0.15). In contrast, within services A and B, there were 40 participants with a sample taken within 48 h of last deployment (subgroup XY in Fig. 1)and for these there was a strong positive correlation between total exposure and 1-HPng/g creat (*r* = 0.53, *P* < 0.001). Better use of RPE during the first deployment was negatively correlated with 1-HPng/g creat (*r* = −0.31, *P* = 0.05) in this subgroup of 40, suggesting a degree of protection, and remained negative but was no longer significant (*P* = 0.33) in a regression that also included exposure.

A second group potentially providing important data comprised those who had been deployed only once at the time the sample was taken. For these, information about the first deployment, particularly the effects of RPE use and skin exposure, were more readily interpretable. In all, there were 77 who reported only a single deployment prior to sample collection, but this included 5 from Service C for whom a single deployment was very unlikely and whose time between exposure and sample collection was too long to be meaningful. Among the 70 from Service A and 2 from Service B, there were 59 (all from Service A) who had been deployed for at least 24 h, who attended the fire strictly as firefighters and not in some logistic or managerial role, were not a current smoker and for whom a sample had been collected within 14 days of the end of their first deployment. In a regression model (Table 4) 1-HPng/g was negatively related to the SEMI score (with better skin hygiene related to lower 1-HP) but not to RPE use. Those who had been deployed to some other fire since returning from Fort McMurray also had higher 1-HP, but estimated exposure and recent BBQ were not found to be important.

**Table 4.** Relation between 1-HP ng/g creat and exposure, RPE use and skin exposure mitigation (SEM) index in non-smokers with one deployment as a firefighter. Multivariable regression analysis allowing for confounding.

	All (*N* = 59)				Deployed in first week (*N* = 20)			
	Beta	SE	95% CI	*P*	Beta	SE	95% CI	*P*
Exposure index	−0.01	0.33	−0.67 to 0.65	0.98	−0.16	0.68	−1.61 to 1.29	0.82
RPE index	3.32	33.70	−64.30 to 70.94	0.92	137.01	95.57	−67.92 to 342.02	0.17
SEM index	−24.98	10.05	−46.14 to −4.82	0.02	−38.15	17.03	−74.66 to -1.63	0.04
Recent BBQ	4.95	16.77	−28.69 to 38.59	0.79	13.59	34.44	−60.27 to 87.45	0.70
Deployed to another fire within last 14 days	41.35	17.79	5.60 to 77.03	0.02	37.43	33.59	−34.62 to 109.48	0.28

A difficulty in interpreting these data was that RPE use and skin hygiene were both negatively related to estimated exposure (RPE: *r* = −0.25, *P* = 0.04: SEMI: *r* = −0.41, *P* = 0.001), reflecting the improved conditions after the first days of the fire when exposure decreased and RPE use and skin hygiene improved. Indeed, the lack of opportunity to change into clean clothes or to shower was reported only by those deployed during the first week of the fire. To examine the effect of this most directly, a final subgroup (subgroup WX) was selected from the 59 firefighters included for the previous analysis. These were 20 firefighters deployed during the first week of the fire (all from Station A). In this analysis, also shown in Table 4, only the SEM index was significantly related to 1-HPng/g creat (*P* = 0.02), having allowed for exposure, RPE use and recent BBQ: exposure to other fires did not contribute.

Within this key subgroup, each component of the SEMI scale showed a clear (although not, for showering, significant) decrease in 1-HP with improved opportunity for cleanliness (Table 5), with no difference seen in any of the other key factors that would confound interpretation: environmental exposure, use of RPE and days between last exposure and sample collection.

**Table 5.** Mean urinary 1-HP ng/g creat, exposure, RPE index, and days between end of exposure and sample collection by skin mitigation factors: non-smokers, deployed as firefighters in the first week of the fire, one deployment to sample collection.

Skin exposure mitigation factor	*N*	1-HPµg/g creat			Exposure index			RPE index			Days to sample		
		Mean	SD	*P*	Mean	SD	*P*	Mean	SD	*P*	Mean	SD	*P*
Able to Change clothes													
No	7	136.8	85.9	0.04	45.38	26.13	0.46	0.25	0.15	0.62	10.14	1.68	0.77
Yes	13	58.7	69.5		53.92	23.36		0.21	0.18		10.38	1.81	
Shower													
No	7	121.7	101.7	0.16	46.35	25.30	0.55	0.23	0.16	0.92	10.71	1.89	0.44
Yes	13	66.8	67.2		53.40	24.00		0.22	0.18		10.08	1.66	
Wash in breaks													
No	9	128.8	105.6	0.03	50.47	28.94	0.94	0.24	0.14	0.85	10.89	1.62	0.17
Yes	11	51.1	33.0		51.31	20.68		0.22	0.19		9.82	1.72	
SEMI score													
0	3	220.8	43.9	<0.01*	52.04	42.29	0.72	0.38	0.13	0.09	11.67	1.53	0.23
1	3	74.0	41.2		35.67	7.77		0.17	0.10		9.00	0.00	
2	8	67.3	87.4		50.99	19.98		0.14	0.05		10.63	1.77	
3	6	49.8	26.8		57.42	26.91		0.30	0.24		9.83	1.84	
Overall	20	86.1	82.7		50.93	24.04		0.23	0.17		10.30	1.72	

*Test for linearity *P* = 0.003.

## Discussion

This paper is, to our knowledge, the first to examine urinary 1-HP in firefighters fighting an uncontrolled fire over many days. Because of the conditions, the design of the study was less than ideal: very few of the urine samples were collected immediately after exposure and none were collected during the first hectic days of the fire. For key analyses, we concentrated only on subgroups in which the time from last exposure to sample collection varied very little. In this way we were able to confirm, in those with short delay in sample collection (subgroup YZ), that 1-HP was related to our estimates of exposure and, even with a mean collection delay of 10 days (range 7–13 days) (subgroup WX), a clear reduction in 1-HP excretion with better opportunities for mitigation of skin absorption.

In interpreting these results, it is important to recognize that the firefighters involved in this analysis of respiratory protection and skin mitigation factors were all from Fire Service A, with overall exposure very much lower than those from Fire Service B and C, and with better access to RPE and skin hygiene measures during the first deployment. They also had the lowest mean 1-HPng/g creatinine. Had we been able to measure urinary 1-HP at the end of the first deployment, we would expect to have obtained much higher concentrations for all firefighters, with highest values for Service C.

The level of 1-HPng/g creat present in these urine samples is low, and within the population distribution for Canada (Health Canada, 2017), in part reflecting the delay (and substantial reduction in body burden) in collecting samples. We only had eight same-day samples, and these were collected in June, long after the full ferocity of the fire. The highest concentration estimated by LC-MS/MS in sample from a non-smoker was from one of these 8 same-day samples. This concentration, 362 ng/l (0.36 µg/l), was far below the suggested BEI of 2.5 µg/l for end-of-week, end of shift sample (ACGIH, 2017). It is also lower than that observed in samples collected immediately after controlled fire by other groups. Adetoma *et al*. (2017) reported mean post-shift levels of 576 ng/g creat in wildland fire fighters. The maximum in a non-smoker here was 272 ng/g creat (log µg/g creat −1.3), at the lower end of the range reported by Keir *et al*. (2017).

The observation that skin exposure was important for these firefighters is concordant with results for heavily exposed industrial workers. Earlier studies, suggesting that up to 90% of exposure may be through the skin, are reviewed by Jongeneelen *et al*. (2001) and more recent studies (Vaananen *et al*., 2005) (McClean *et al*., 2007) have confirmed the importance of this route for asphalt workers. In studies of firefighters, skin wipes following prescribed burns have confirmed the presence of PAHs on the skin (Fent *et al*. 2014; Fernando *et al*., 2016; Keir *et al*., 2017; Stec *et al*., 2018). There has also been concern about the extent to which contamination of firefighters’ turn-out gear (Fent *et al*. 2017), protective equipment (Stec, 2018) or the indoor air of fire stations (Oliveira *et al*., 2017) contribute to PAH exposure after return from active firefighting. In the present study, the 1-HP levels in firefighters from Service C (whose last deployment was at least 37 days before sample collection) may have resulted in part from on-going residence in a city in which the smell of smoke was still present at the time of sample collection and all surfaces were potentially contaminated.

In talking to the firefighters, use of RPE was at best intermittent during the early days of the fire. There was no standard issue of equipment and many found themselves without masks or spare filters. Even where they were available, they were often not worn in the first frantic phase of the fire as they were felt to interfere with communication, respiration during heavy physical work or became so rapidly contaminated as to be useless. The RPE index computed to reflect factors affecting the effectiveness of protection has not been validated and should be taken only as an indication of how poor protection was in the first days of the fire, with overall, only 16% protection, based on self-reports of the proportion of time worn, how often it was changed and the published protection factor for the type of RPE.

Analysis and interpretation of this dataset has been challenging: we found intractable confounding between length of time from last exposure to sample collection and estimated exposure, which was circumvented only by stratification. The interpretation of patterns in 1-HP concentrations at low levels in urine samples collected beyond the fast excretion half-life is also problematic, as the possibility of unmeasured confounding, for example by diet (beyond recent BBQ) or secondhand tobacco smoke, must also be considered: such factors could cause small changes in 1-HP concentration such as seen here, but to explain the differences in Tables 4 and 5 they would have to be related to the SEMI (the availability of skin hygiene measures during the first week of the fire). Such confounding seems less likely, given the body of literature on skin absorption of PAHs, than the slow-phase excretion, at low levels, of PAHs absorbed, in part, through the skin in those with poor skin hygiene.

Finally, it is worth considering how far such observational rather than experimental studies can contribute to knowledge of the relation between exposure, excretion and potential control. It is notable, in the area of PAH exposure, the extent to which important conclusions have been drawn from non-experimental observations on individual workers. Jongeneelen *et al*. (1988) followed a single operator of a creosote impregnating plant during a prolonged period away from work and found a bi-phasic excretion pattern (interpreted by ACGIH (2017) as tri-phasic), with a 16-day half-life for the second phase. Lafontaine *et al*. (2000) first observed the longer half-life with skin absorption in a single worker and absorption on non-exposed days through contaminated overalls (Lafontaine *et al*., 2002) before setting up experimental work to examine the relation between skin absorption and excretion *in vitro* and in rats (Payan *et al*. 2008). The interpretation of the current data depends heavily on these (non-experimental) observations, and particularly on the late phase of excretion reported by Jongeneelen *et al*. (1988) and supported through experimental exposures by Viau *et al*. (1995) with the same group demonstrating late excretion in experimentally exposed rats (Bouchard *et al*., 2002). While these later phase half-lives may be irrelevant to setting exposure standards, they are critical to the interpretation of the data presented in this paper.

The difficulties in analyzing and interpreting results in this study underline the need for rapid collection of biological samples to assess exposures during such an emergency. Urine samples are easily collected, and I-HP is stable over many months, without elaborate or costly storage facilities (Jongeneelen *et al*., 1987). Although handicapped by the absence of such timely samples, the results from this study demonstrate the importance of reducing possibilities of skin absorption (with ingestion as a possible concomitant route). While this is becoming widely recognized for firefighters in station-based engagements (Fent *et al*. 2017; Wingfors *et al*., 2018), the message is no less important for firefighters battling on the ground to contain a highly destructive fire such as that in Fort McMurray.

## Supplementary Material

Supplementary Material
