# Supplementary material for: Urinary 1-hydroxypyrene and Skin Contamination in Firefighters Deployed to the Fort McMurray Fire
Source: Ann Work Expo Health. 2019 Feb 8;63(4):448–58. doi: 10.1093/annweh/wxz006 (PMC6473170; doi:10.1093/annweh/wxz006)
Supplement: Supplementary Material [file wxz006_suppl_supplementary_material.pdf]

Online supplementary materials.

Urinary 1-hydroxypyrene and skin contamination in firefighters deployed to the Fort McMurray fire.

Nicola Cherry[1] Yayne-abeba Aklilu[2], Jeremy Beach[1], Philip Britz-McKibbin[3], Rebecca Elbourne[1], Jean-Michel Galarneau[1], Biban Gill[3], David Kinniburgh[4], Xu Zhang [4]

1. Division of Preventive Medicine, University of Alberta, Edmonton, Alberta, Canada
2. Alberta Environment and Parks, Edmonton, Alberta, Canada
3. Department of Chemistry and Chemical Biology, McMaster University, Hamilton, Ontario, Canada
4. Alberta Centre for Toxicology, Calgary, Alberta Canada.

Supplementary materials 1: Instrumental Analysis (LC-MS/MS method)

1. LC-MS/MS

LC-MS/MS analysis was performed using an Agilent 1200 high performance liquid chromatograph (Agilent Technologies, Santa Clara, CA, USA) coupled with a Sciex 5500 Q-trap mass spectrometer (AB Sciex, Concord, Ontario, Canada). Separation of 1-hydroxypyrene was achieved on an Agilent SB-C18 column (Poreshell 120, 3.0X100 mm, 2.7µm) using isocratic elution of water (40%) and acetonitrile (60%) at a flow rate of 0.4 ml/min. The injection volume was 10 µL and the column was kept at room temperature. The mass spectrometer was operated in negative MRM mode. The source temperature was 550 °C with ion spray potential of -4500v, curtain gas at 20, gas 1 at 45, gas 2 at 55 and CAD at high. The MRM transition for the detection of 1-OH-pyrene was 217.0/188.9 with collision energy (CE) at -45 and declustering potential (DP) at -140. The MRM transitions for 1-OH-Pyrene-d9 (ISTD) was 226.1/198.1 with CE at -48 and DP at -150. Data processing was conducted using Multiquant3.0.1 (AB Sciex, Canada).

2. Reagents and Sample Preparation

Reagents, including acetonitrile (HPLC grade), sodium acetate (reagent grade, >99%), L-Ascorbic acid (≥99%), Glacial acetic acid (HPLC grade), Potassium dihydrogen phosphate (ACS reagent, ≥99%), Surine® negative urine, and β-glucuronidase (from Helix pomatia, Type HP-2, aqueous solution, >100,000 units/ mL) were purchased from Sigma-Aldrich (Canada). 1-hydroxypyrene (1-OH-pyrene), and 1-hydroxypyrene-d9 (1-OH-pyrene-d9) were purchased from Toronto Research Chemicals Inc. (Canada). Stock solutions of standards were prepared by dissolving in acetonitrile to a concentration of 1 mg/mL and then further diluted in acetonitrile using serial dilutions.

Sample preparation involves an enzyme deconjugation, followed by protein precipitation and dilution. To an aliquot of 1 mL of urine, 5 µL of ascorbic acid (1.5M), 10 µL of internal standard (1-OH-pyrene-d9, 100 ng/mL), 200 µL of pH5.2 buffer and 10 µL of diluted β-glucuronidase/sulfatase were added. The mixture was incubated on a shaking water bath for 2 hours at 60 °C and then cooled to room temperature. 100 µL of hydrolyzed sample was aliquoted and further diluted with 5uL of ascorbic acid and 900 µL of elution solvent (ACN/pH6 buffer, 0.1M, 2:1)). The mixture was vortexed and then centrifuged at 4000 rpm for 10 min. The supernatant was transferred to a 2 mL silanized autosampler vial for LC-MS/MS analysis.

3. Quality control and Data Analysis

A set of matrix matched calibrators were prepared by spiking known amounts of 1-OH-pyrene into Surine® negative urine from 0.02 ng/mL to 10 ng/mL. Two in-house QCs were prepared at 0.1 ng/mL and 0.5 ng/mL by a different analyst. A standard reference material® (SRM 3673) which contains 0.029 ng/mL of 1-OH-pyrene in urine was purchased from National Institute of Standards & Technology (NIST) and used as an outsource QC. Each batch consisted of 40 Fort McMurray samples, a set of calibrators, a solvent blank, a urine blank, two in-house QCs and SRM 3673. All blanks, calibrators and QCs were hydrolyzed and cleaned up along with the samples. The Blanks and QCs were run after calibrators, in the middle of the sequence and at the end of sequence to insure all samples were bracketed with blanks and QCs. QCs must be within

±20% of targeted value. The quantification of 1-OH-pyrene in the samples was calculated based on the peak area ratio of 1-OH-pyrene to internal standard in the sample against the calibration curve constructed from the calibrators. The retention time of 1-OH-pyrene in the sample must be within ± 0.1min of that in the calibrators. The batch was accepted when all QCs passed and all the blanks were clean.

4. Method validation

Surine® artificial negative urine was used to validate the method because blank urine from non-smokers may still contain trace levels of 1-OH-pyrene. Method validation was conducted by spiking Surine® negative urine with known amounts of 1-OH-pyrene and 1-OH-pyrene-d9 as internal standard, then hydrolyzed and cleaned up following the procedure described above. It was observed that 1-OH-pyrene was linear up to 500 ng/mL with a correlation coefficient (R^2) greater than 0.995. Fig.1 shows a representative calibration curve from 0.02ng/mL to 10 ng/mL. The limit of detection (LOD) and limit of quantification (LOQ) were defined as the concentration of 1-OH-pyrene at which the signal-to-noise ratio (S/N) is equal to or greater than 3 and 6. The method LOD and LOQ are 0.01 ng/mL and 0.02ng/mL respectively. Fig 2 depicts representative chromatograms of the internal standard and 1-OH-pyrene in a human urine sample.

It is well known that LC-MS/MS is prone to ion suppression or enhancement due to the presence of matrix components. Matrix effect (ME) was evaluated in blank urine from different volunteers. Ion suppression was less than 10%.

Recovery, accuracy and precision of spiked urine samples was performed at concentrations of 0.1 ng/mL, 1 ng/mL and 10 ng/mL. The results were summarized in the table below.

1-OH-pyrene in urine (ng/mL)	Recovery (%)	Accuracy (%)	Precision (CV %)
0.1	88.2	95.2	7.4
1	93.2	103	0.65
10	97.5	99.7	0.52

NIST SRM® 3673 is a urine based certified reference material which contains 0.0293ng/mL of 1-OH-Pyrene. It was used to evaluate the accuracy at near LOQ. The accuracy was 88.1% with coefficient of variation (CV) of 5.1%.

Fig. 1. Calibration curve for 1-hydroxypyrene up to 10 ng/mL

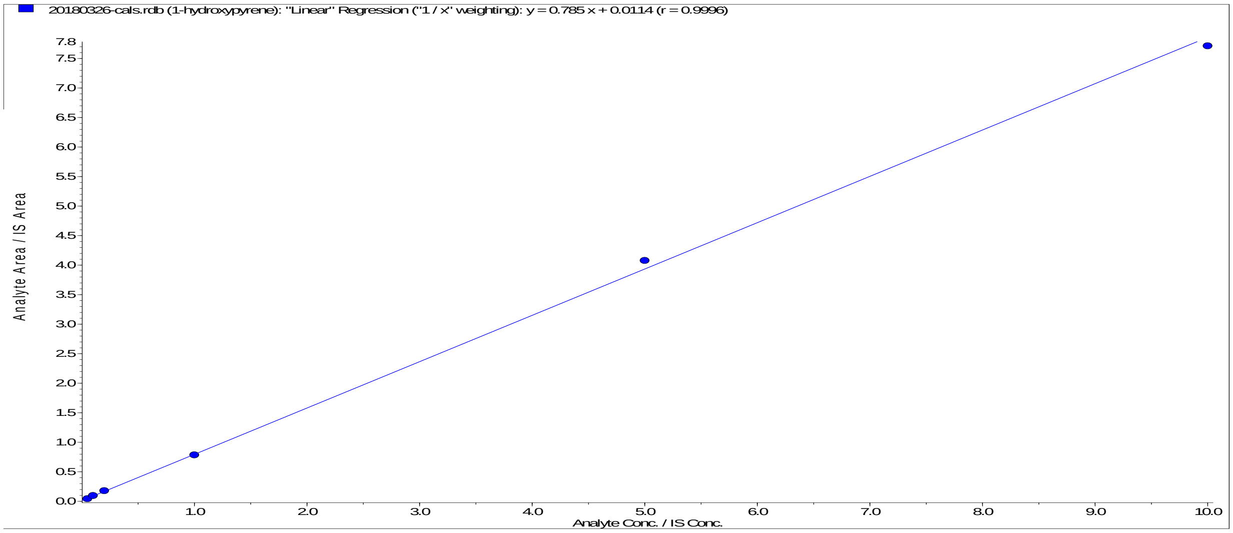

Fig. 2. LC-MS/MS MRM chromatograms of 1-hydroxypyrene and 1-hydroxypyrene-d9 (ISTD) in human urine

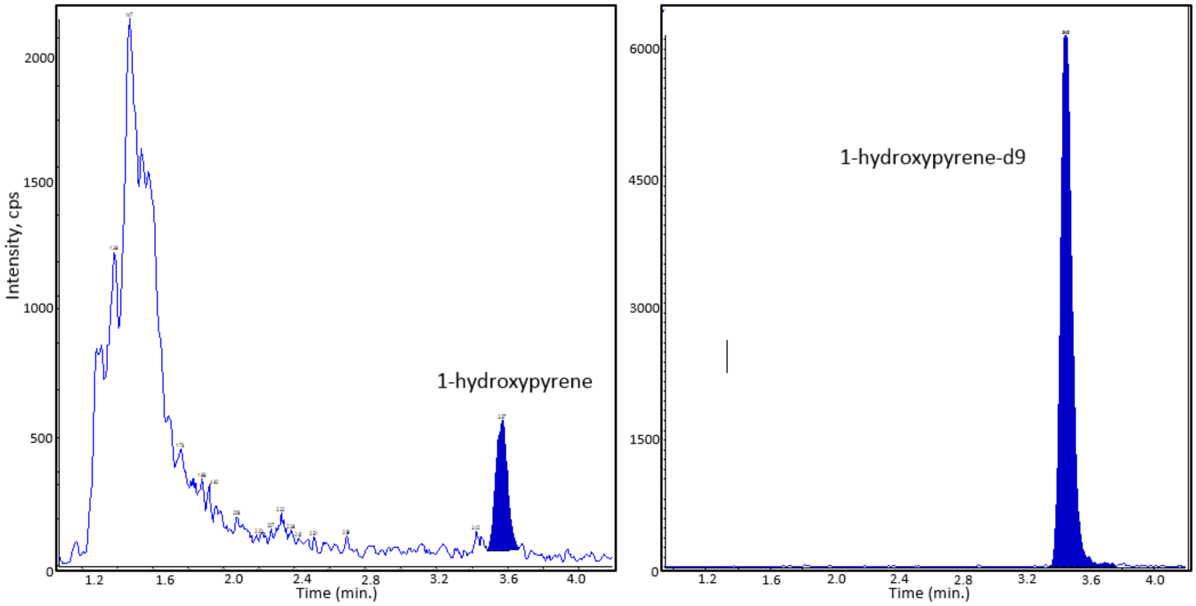

Supplementary materials 2

A) Estimation of environmental smoke concentration (total PM2.5) encountered by each firefighter during the key rotation.

Potential exposures were calculated for the 'key' deployment. This was the first deployment for all but 4 firefighters from fire station A, who gave information only for their most recent deployment. Elements used for this calculation were as follows

1) Data available from Alberta Environment and Parks

Daily 24-hour average concentrations of $PM_{2.5}$ ($\mu g/m^3$) for each day from May 1st 2016 to June 30th 2016 were provided for 9 locations within the urban area of Fort McMurray. These had been listed in the questionnaire and the firefighter was requested to indicate the percentage of time spent in each. These were:

Timberlea, Thickwood, Abasand, Lower Town Site, Waterways, Beacon Hill, Gregoire, Saline Creek, Parson's Creek.

Estimates were also obtained for 3 further locations (the airport, the village of Anzac and the area of Mildred Lake) as these were reported by firefighters as additional areas in which they spent time during their key deployment. All estimates except those for Mildred Lake used data from Alberta Environment monitoring stations. The estimates for Mildred Lake use Blue Sky estimates (a combination of air samples taken at different locations and satellite imagery).

2) Total hours on active duty reported by the fire fighter during the key rotation.

The number of hours worked on each day of each shift during the key deployment was calculated.

For example: for firefighter 000 shift 1 started at 9h00 on May 3rd with reported length 23 hours; he will have worked from 9h00 to 23h59 on May 3rd so 15 hours for May 3rd and he would have worked 9 hours on May 4th. This was repeated for every shift as to determine how many hours were worked on what days for their entire key deployment.

3) The percentage of time spent in each location during the key deployment (as reported by the firefighter)

This was used to compute a time weighted exposure for each firefighter for each day of deployment.

For example: firefighter 000 worked on May 3rd 10% in Parson's Creek and 80% in Timberlea for 5 hours. Alberta environment has a 24-hour mean for Parson's Creek on May 3rd of 535 $\mu g/m^3$ and of 320 for Timberlea (535*5*.1) + (320*5*.8).

4) The total time was used to adjust the time weighted estimate

PM2.5 estimates were adjusted to reflect 100% of their day because many firefighters either overestimated or underestimated the amount of time they spent in each location (i.e the total percentage exceeded or fell short of 100%)

Example: (535*5*.1) + (320*5*.8)=1547.5 for 90% of key deployment adjusted as 547.5/0.9=1719.44

5) A cumulative exposure for their key deployment was obtained by summing over all shifts

B) Calculation of total exposure to $PM_{2.5}$ across multiple deployments

1) For all firefighters that had multiple deployments to Fort McMurray between May 1st and June 30th 2016, no specific information as to where they worked and how much time they spent in each area was obtained for deployments other than the key rotation. Additional exposures were therefore estimated from the cumulative estimates made for key deployments across all firefighters.

2) To do this their cumulative estimates were divided into attributable portions for each day worked during their secondary deployments. This was done using the estimates calculated on each day in Step 1. A mean exposure for each date worked as a key deployment was obtained. Data was available for almost every day in their deployments (between May 1st to June 30th), however, there were dates, mostly in June, for which no data was available due to the very low or non-existent numbers of respondents initially deployed during those dates. To replace those missing estimates, data from Alberta Environment as a whole was used to calculate the means for each of those days and those were multiplied by 10 hours, approximately the mean number of hours worked for firefighters working in late May and June.

3) This equals a per deployment estimate. These were summed to obtain a cumulative exposure during all non-key deployments and then added to the estimated exposure during the key deployment to estimate total exposure across all deployments.

Supplementary materials 3: Construction of the RPE index

- The reciprocal of the protection factor of the best RPE equipment identified was computed (protection factor of 10=1/10 unprotected).
- The recommended time to change of a cartridge was taken as 5 hours (reflecting a reduction from 8 hours due to heavy exposures) to thus the total time spent in each task was divided by 5 to produce a number of times *should have changed*. The time in each task was divided by the number of hours to actual change producing number of times actually changed.
- Ratio of *times changed* to *should have changed* was computed. This ratio was multiplied by the total time wearing respirator in each task to produce a time improperly wearing RPE which was added to the actual time not wearing RPE (when ratio<1.0). This time was treated as unprotected.
- The reciprocal of the RPE was used and multiplied by the remaining properly protected time.
- Both of these values were added giving us a value between 0 and 1 which represents an unprotected time factor (1=unprotected, 0=100% protected).
- This was cumulated over all tasks and divided by the number of tasks and adjusted to reflect 100% of their time during their key deployment.
- The final score was rescaled such that zero indicated no protection and 1 perfect protection
